# Data driven flexible backbone protein design

**DOI:** 10.1371/journal.pcbi.1005722

**Published:** 2017-08-24

**Authors:** Mark G. F. Sun, Philip M. Kim

**Affiliations:** 1 Department of Computer Science, University of Toronto, Toronto, Canada; 2 Terrence Donnelly Centre for Cellular and Biomolecular Research, University of Toronto, Toronto, Canada; 3 Department of Molecular Genetics, University of Toronto, Toronto, Canada; 4 Banting and Best Department of Medical Research, University of Toronto, Toronto, Canada; University of Oxford, UNITED KINGDOM

## Abstract

Protein design remains an important problem in computational structural biology. Current computational protein design methods largely use physics-based methods, which make use of information from a single protein structure. This is despite the fact that multiple structures of many protein folds are now readily available in the PDB. While ensemble protein design methods can use multiple protein structures, they treat each structure independently. Here, we introduce a flexible backbone strategy, FlexiBaL-GP, which learns global protein backbone movements directly from multiple protein structures. FlexiBaL-GP uses the machine learning method of Gaussian Process Latent Variable Models to learn a lower dimensional representation of the protein coordinates that best represent backbone movements. These learned backbone movements are used to explore alternative protein backbones, while engineering a protein within a parallel tempered MCMC framework. Using the human ubiquitin–USP21 complex as a model we demonstrate that our design strategy outperforms current strategies for the interface design task of identifying tight binding ubiquitin variants for USP21.

## Author summary

To engineer proteins that bind a target protein, computational protein design methods have been employed to reduce the set of candidate protein variants that are then experimentally validated. Current protein design methods use protein backbone information from a single protein structure. In order to use alternative protein backbones, the single input protein structure is perturbed in either a random manner or restricted to a predefined set of protein backbone movements. Here, we introduce the FlexiBaL-GP protein design strategy, which incorporates information from multiple protein backbones to guide the search for new alternative high quality protein backbones. These large multi-atom movements of a protein system are learned via the non-linear dimensionality reduction method provided by Gaussian Process Latent Variable Models (GP-LVM). Using the GP-LVM approach, our strategy jointly searches for new protein backbones and new protein variants. We demonstrate on the ubiquitin–USP21 system that the FlexiBaL-GP design strategy outperforms current protein design approaches.

## Introduction

Protein engineering has created new proteins as therapeutics [1–3], modulators of protein interactions [4,5] or to enhance enzymatic activity [6–9]. Experimental directed evolution approaches have been quite successful at identifying tight binding protein variants from a large pool of randomized variants [10–12]. On the other hand, computational approaches search over the large space of possible amino acid combinations to identify a short list of protein variants likely to exhibit the function of interest. While they can search many more variants than experimental approaches, often only a small proportion of the short- listed protein variants are experimentally found to tightly bind the target of interest. This difficulty can be attributed 1) to approximations made by the scoring functions used to rank different protein variants and 2) by the search methods used to explore the rugged energy landscape of a protein fold [13].

Computational protein design strategies typically attempt to identify the optimal sequence for a protein fold, by searching over alternative residue side chains and protein backbones. Two general protein design approaches exist, ensemble backbone design and flexible backbone design, which differ in how the protein backbone is explored. Ensemble backbone design strategies employ a two-phase approach, where multiple protein backbones composed of the same sequence are first created. The optimal sequence is subsequently identified independently for each protein backbone within the ensemble using a simulated annealing or dead-end elimination approach [14,15]. Flexible backbone design methods explore different protein backbones and amino acid combinations within the same procedure. In this manner, alternative protein backbones composed of different amino acid combinations can be explored. By considering alternative backbone configurations, ensemble and flexible backbone strategies are able to identify protein variants that would otherwise be omitted if a single backbone template were used. Together, these methods have successfully identified protein variants with enhanced binding affinity for a target or better thermostability, in addition to capturing a protein’s binding specificity [14–19]. Recent advances have led to methods that link such computational design methods with high throughput screening methods leading to improved designs [20].

**Fig 1:**
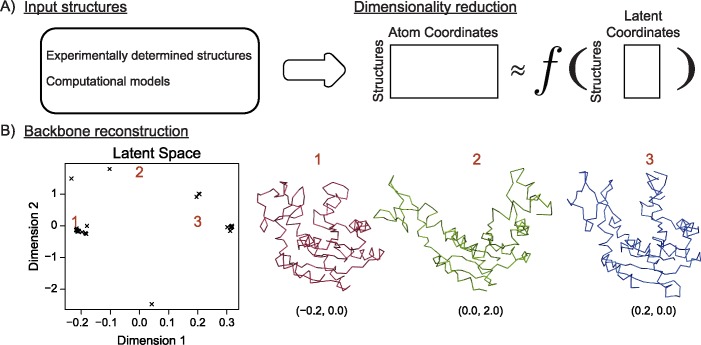
Schematic of incorporating multiple protein structures for protein design. A) Multiple structures are aligned and a non-linear dimensionality reduction method maps the atomic coordinates into a 2 dimension latent space. B) Traversals in a latent space, designated by points 1, 2, and 3, corresponds to complex multi-atom movements, where x denotes the location of an input structure.

Exploring alternative protein backbones is currently achieved by applying perturbation operations on a single protein structure. Protein backbone perturbation operations have included the random displacement of backbone torsion angles [21], sampling from a predefined set of short structural configurations, sampling from a predefined set of previously observed backbone movements such as shearing and the “backrub” rotation movement [17,22,23], or sampling along movement trajectories defined by normal mode analysis [24]. None of these approaches take advantage of the fact that, increasingly, there are multiple structures of the same protein fold (or, indeed, the same protein) available at the protein data bank (PDB). Here we sought to incorporate information from multiple protein structures using machine learning. This is achieved by performing a non-linear dimensionality reduction procedure over the atomic coordinates of multiple protein structures. In doing so, we directly learn complex protein backbone movements. We incorporate these learned backbone movements in a parallel tempering sampling scheme to identify tight binding protein variants. This protein design strategy is called Flexible Backbone Learning by Gaussian Processes (FlexiBaL-GP) design (Fig 1).

We demonstrate our FlexiBaL-GP design strategy in the ubiquitin system, which uses post translational modifications to regulate protein abundance in the cell [25]. It is implicated in many cellular processes and diseases such as cancer [26]. As a proof of concept we sought to first recapitulate the natural conformational ensemble of ubiquitin. Second, we modulate the ubiquitin system by inhibiting ubiquitinase specific protease 21 (USP21), a deubiquitinating enzyme known to regulate cancer associated pathways [27]. Here we use the ubiquitin–USP21 complex as our model and evaluate the FlexiBaL-GP design method by its ability to identify ubiquitin variants that tightly binds USP21. For this purpose, a short contiguous 18 amino acid region on the ubiquitin interface was engineered (positions 54–71). FlexiBaL-GP design is implemented within the Rosetta protein design framework and we demonstrate its ability to identify more ubiquitin variants that tightly bind USP21 than existing ensemble and flexible backbone strategies.

## Results

### Learning flexible protein backbone movements

To account for protein flexibility, current flexible backbone design methods predefine rigid protein movements that can be utilized during the design procedure. Rather than applying general backbone movements, system specific global backbone movements can be directly learned from multiple structures of the same protein. In an ideal scenario, many different structures with all possible conformations would be available, but as we show below, even using only a few structures leads to marked improvements. To do so, we first note that protein backbone atoms are highly constrained by intramolecular and intermolecular forces (often expressed as molecular forcefields). These local restrictions couple the movements of neighboring and spatially close atoms, limiting the number of feasible movements that a protein system can undergo. If all N atoms of a protein system were to move independently, the system would exhibit 6N degrees of freedom, considering translation and rotation operations in Cartesian space, whereas a rigid protein would only have 6 degrees of freedom. Since protein atoms are unlikely to move fully independently, the true number of degrees of freedom is likely much smaller than 6N and we can employ a non-linear dimensionality reduction approach to learn these hidden dimensions, representing protein movements involving correlated atomic movements. In doing so, global atomic movements required to transform one input structure into another can be directly learned from multiple protein structures.

Our FlexiBaL-GP method uses a Gaussian process latent variable model (GP-LVM) to perform a non-linear dimensionality reduction over protein backbone coordinates. The GP-LVM uses a similarity function *k*(⋅,⋅), called a kernel, to specify the relationship between the input protein structures within the lower dimensional space defined by the reduced number of degrees of freedom (Fig 1A). Parameters to be learned within the GP-LVM framework are the locations of the N protein backbones in this reduced space of L dimensions or latent space (**X** ∈ ℝ^*N*×*L*^) and the kernel parameters. If a linear kernel is chosen, the resulting GP-LVM can be thought of as performing probabilistic principal component analysis on the protein backbone atomic coordinates and selecting the first L principal components to represent the protein backbones. Specifically, the GP-LVM relates a latent space (**X** ∈ ℝ^*N*×*L*^) to a data space (**Y** ∈ ℝ^*N*×*D*^) via the mapping *y*_*nd*_ = **f**_*d*_(**x**_*n*_) + *ϵ*_*n*_, for the n’th data point and d’th feature with a Gaussian noise error term, *ϵ*_*n*_ ∼ *N*(0,*β*) with a precision β [28,29]. The GPLVM joint probability model can then be defined as follows after marginalizing the mappings to give

$$\begin{array}{l} p(Y,X|\theta ,\beta )\;=p(Y|X,\theta ,\beta )p(X)\\ \;=\prod_{d=1}^{D}N(y_{d}|0,K+\beta I)\prod_{n=1}^{N}N(x_{n}|0,I). \end{array}$$

For this study, we used the composite kernel constructed from the radial basis function (RBF) and bias kernels, such that

$$k(x_{s},x_{t})=\theta_{0}exp(-\frac{1}{2}\sum_{l=1}^{L}\theta_{l}||x_{s}-x_{t}|{|}^{2})+\theta_{3}.$$

Learning the location of the data points in latent space (**X**), the kernel parameters (**θ**), and noise parameter (β) can be achieved by optimizing the GPLVM joint distribution by minimizing the negative log posterior

$$L(X,\theta ,\beta )=\frac{DN}{2}log2\pi +\frac{D}{2}log|K|+\frac{1}{2}Tr(K^{-1}YY^{T})+\frac{1}{2}\sum_{n=1}^{N}x_{n}^{T}x_{n}.$$

Importantly, once the L dimensional protein backbones representations are learned, new protein backbones sampled from this L dimensional space can be reconstructed in Cartesian space by means of the mapping provided by the GP-LVM (Fig 1B). Of course, because the latent space is of much lower dimensionality, this will be computationally much more efficient than deciding a protein’s movement in Cartesian space. Thus a MCMC sampling strategy can be employed to sample new protein backbones from this L dimensional space, rather than directly perturbing protein backbones in Cartesian space with rigid movement operations. Due to the non-linearity introduced by the described composite kernel, linear traversals in latent space correspond to concerted multi-atom protein backbone movements. In this manner, complex protein backbone can be explored, while searching for alternative amino acid combinations over the designed protein positions (Fig 1B). Reconstructing the atomic coordinates of a new protein backbone, y*, associated with a latent point, x*, is made possible by computing the predictive mean of the conditional Gaussian distribution,

$$p(y^{*}|x^{*},Y,X,\theta ,\beta )∼N(E[y^{*}],\;\mathrm{cov}(y^{*})),$$

where

$$E[y^{*}]=E[\mu_{x^{*}}]+k_{x^{*},X}[K_{X,X}+\beta I{]}^{-1}y_{d}$$ (1)

$$\mathrm{cov}(y_{d}^{*})=k_{x^{*},x^{*}}-k_{x^{*},X}[K_{X,X}+\beta I{]}^{-1}k_{X,x^{*}}.$$

Here, we have used **K**_**X,X**_ and **k**_x*,**X**_ to respectively indicate the kernel matrix from the N input structures and the row vector arising from applying the kernel function between x* and every N input structures.

### Sampling the energy landscape

The energy landscape of a protein fold is thought to be rugged, containing multiple local minima, which may partly be reflected in latent space. If a single MCMC trajectory were utilized at a low temperature to explore alternative protein backbone configurations and protein sequences, the trajectory would get trapped in one of the many local minima. This is akin to executing a single Rosetta Backrub procedure at a very low temperature. Alternatively, if a high temperature were used for the MCMC trajectory, undesirable high-energy solutions may be proposed. We overcome the aforementioned problems while searching for alternative protein backbone configurations and protein sequences, by employing a parallel tempering or replica exchange MCMC sampling strategy. Here, multiple MCMC chains are executed independently at predefined temperatures following a temperature ladder. At each iteration, a state swap between a random chain and a neighbouring chain occurs with a probability determined by the Metropolis-Hastings acceptance criteria. Specifically, protein structures associated with chain *i* and *j* respectively having energies *E*_*i*_ and *E*_*j*_ and temperatures *T*_*i*_ and *T*_*j*_, are swapped with probability:

$$p(i,j)=min(1,exp[(E_{i}-E_{j})(\frac{1}{T_{i}}-\frac{1}{T_{j}})]).$$

Here, we employed 24 MCMC trajectories spanning a temperature ladder defined by a geometric series with a multiplying ratio of 1.1. The lower and upper temperature bounds were respectively set to 0.1 and 0.5.

### A global flexible backbone strategy

Our proposed flexible backbone protein design strategy, FlexiBaL-GP, incorporates learned protein backbone movements within a parallel tempering framework. As input, D atomic coordinates of heavy atoms (∈{N, Cα, C, and O}) from N structurally aligned protein backbones is provided to construct the data space (**Y** ∈ ℝ^*N*×*D*^). First, the GP-LVM parameters are learned using the GPy framework [30], which includes the location of the N input protein structure in latent space (**X** ∈ ℝ^*N*×*L*^), where L is the dimension of the reduced space (L = 2 in this study). Second, a parallel tempering strategy is used to explore alternative amino acid combinations and protein backbones. At each iteration, a new amino acid side chain rotamer or a new protein backbone is respectively chosen 95% and 5% of the time. Amino acid side chains are sampled from the Dunbrack 2010 rotamer library, where each amino acid is considered equiprobably. Proline rotamers are omitted due the Rosetta protein design framework’s representation of amino acid side chains. New protein backbones are sampled from the 2 dimensional latent space defined by the GP-LVM, using a zero-mean univariate Gaussian to determine the latent space step. The Gaussian variance is set to 0.001 times the maximum difference between the N input structures in the latent space for each latent dimension. The new sampled protein backbone space is subsequently reconstructed in Cartesian space using Eq 1. For the protein backbone movement, the newly reconstructed protein retains the same side chain rotamers as the one before the backbone movement operation. Since all protein backbone heavy atoms may have moved, the backbone hydrogen atoms are rebuilt into the new protein backbone configuration. Once either a rotamer or backbone change has been formed, the new change is accepted with a probability determined by the Metropolis-Hastings acceptance criteria. Note that reconstructing the protein backbone with a Gaussian process is relatively fast, due to the small number of required matrix operations; however, hydrogen and side chain placements over the reconstructed backbone are computationally more expensive operations. Thus we use a partial structure, defined by an 8Å distance threshold around the engineered positions, which reduces the computational time required to perform these flexible backbone movements. Together, the described procedures form the FlexiBaL-GP protein design strategy, which we implement in C++ within the Rosetta protein design framework. OpenMP is used to take advantage of the MCMC trajectories that are independently executed with the parallel tempering framework. Since parallel tempering methods sample from the target distribution after equilibration, the scores of multiple poses associated with the same amino acid sequence are merged following the Boltzmann distribution function, $\sum_{p}^{N}e^{-E_{p}}$, where *E*_*p*_ is the Rosetta energy score for pose *p* of a given sequence.

### Recapitulating the natural ubiquitin ensemble

**Fig 2:**
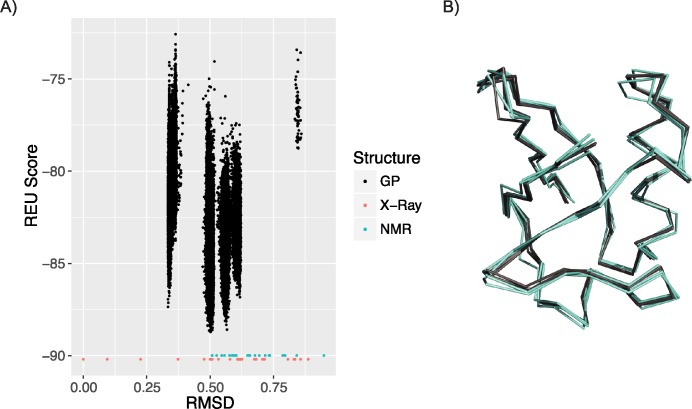
Unbound ubiquitin ensemble. A) RMSD vs Rosetta energy unit (REU) score for the 10 lowest temperature trajectories of a single FlexiBaL-GP execution (black), 28 X-ray crystal structures (red), and 25 randomly selected NMR models (cyan). B) Superimposition of 3 randomly selected FlexiBaL-GP models (black) and 3 randomly selected NMR models (cyan).

We first assessed the FlexiBaL-GP method by its ability to capture the natural conformations of ubiquitin. Using 28 X-ray crystal structures as input, we asked if the FlexiBaL-GP method could recapitulate the ubiquitin backbone conformation variation observed amongst the NMR models (PDB id 2LJ5) [31]. The majority of low energy models derived from the FlexiBaL-GP method are within 0.50–0.65 Å RMSD of the 1UBQ reference structure. The 25 randomly selected NMR models were found to have a greater diversity, spanning 0.509–0.949 Å RMSD to the reference structure, with 44% of the NMR models located within the range spanned by the FlexiBaL-GP models (Fig 2A). Visual inspection of 3 randomly selected FlexiBaL-GP models derived from the lowest temperature trajectory and 3 random selected NMR models illustrates that the FlexiBaL-GP method can partially capture the natural ubiquitin conformational ensemble. More interestingly, it demonstrates that just 2 latent dimensions are sufficient for the construction of natural ubiquitin structures (Fig 2B).

### Designing ubiquitin variants for tight binding towards USP21

Next, the FlexiBaL-GP method is assessed by its ability to design the ubiquitin interface (positions 54–71) for tight binding towards USP21. We and others have previously redesigned ubiquitin for binding to USP21 [20,32] and have thus obtained 215 experimentally validated tight ubiquitin variants. We have previously shown that a random forest regression model trained on these can accurately predict affinity for any ubiquitin variants [20]. We used this regression model to determine which variants identified by the FlexiBaL-GP approach are likely to tightly bind USP21 and to compare FlexiBaL-GP to other design methods. To evaluate the impact of varying the number of protein backbone structures from different sources, we apply the FlexiBaL-GP strategy to three input scenarios: 1) two wild type complexes (PDB id 3I3T and 2Y5B), 2) two wild type complexes augmented with molecular dynamic models, and 3) two wild type complexes and a complex of a variant previously found to tightly bind USP21 (PDB id 3MTN) [32,33]. For scenario 2, 20 structures in total are used as input, composed of 2 wild type crystal structures (PDB id 3I3T and 2Y5B) and 18 models derived from 100ns molecular dynamic simulations initialized using the 3I3T and 2Y5B structures (9 models each). Molecular dynamic models were employed to expand upon the space of possible protein backbones of the ubiquitin–USP21 system. For the following comparisons, the FlexiBaL-GP method was executed 5 times for each scenario to ensure stability of the employed sampling scheme.

We compared the FlexiBaL-GP approach to conventional protein design strategies of ensemble and flexible backbone design to engineer the ubiquitin interface for tight binding towards USP21. Two ensemble approaches were used for the creation of ubiquitin–USP21 complex ensembles, 1) molecular dynamics [34,35] and 2) CONCOORD, a distance constraint approach [36]. The multi-cooling Rosetta simulated annealing procedure was used to design the ubiquitin interface for USP21 binding for each structure within the ensemble. The Rosetta Backrub method was chosen as a representative flexible backbone design method. Variants arising from the described ensemble and flexible backbone design methods were retrieved from our previous study that all used the 3I3T structure as input [20]. For all design strategies, the talaris2013 Rosetta scoring function was used and the top 2000 scoring variants were extracted for the comparisons. In this manner, differences in the ubiquitin variants generated from each design strategy can be attributed to the search strategy employed by a design method for the discovery of alternative side-chain and backbone configurations.

We first assessed the design strategies by their ability to recover variants that were similar to experimentally identified tight binding ubiquitin variants for USP21 using a high throughput screening method [32]. As different protein positions were selected for engineering compared to the experimental method, only overlapping ubiquitin positions (62, 63, 64, 66, 68, 70, and 71) were compared. For each designed sequence, the closest matching experimentally identified variant was found from which the sequence identity was computed. Taking the mean over the maximum sequence identities for each design strategy, we find that all FlexiBaL-GP scenarios, the designed sequences were more similar by sequence identity to the experimentally identified sequences compared to sequences derived from either ensemble or flexible protein design strategies. Specifically, FlexiBaL-GP scenarios 1, 2, and 3 respectively achieved an averaged maximum sequence identity of 53.55%, 57.89%, and 53.52%, while the MD ensemble, CONCOORD ensemble, and Backrub design methods achieved an averaged maximum sequence identity of 44.42%, 47.55%, and 44.94%. Focusing on position 68, which was deemed to be instrumental for tight binding for USP21, 98.35%, 89.95%, and 98.4% of the variants respectively from FlexiBaL-GP scenarios 1, 2, and 3 contained the phenylalanine or tyrosine amino acids [32]. This contrast with 36.75%, 51.65%, and 29.10% of the variants respectively from the MD ensemble, CONCOORD ensemble, and Backrub design methods that had either a phenylalanine or tyrosine at position 68.

**Fig 3:**
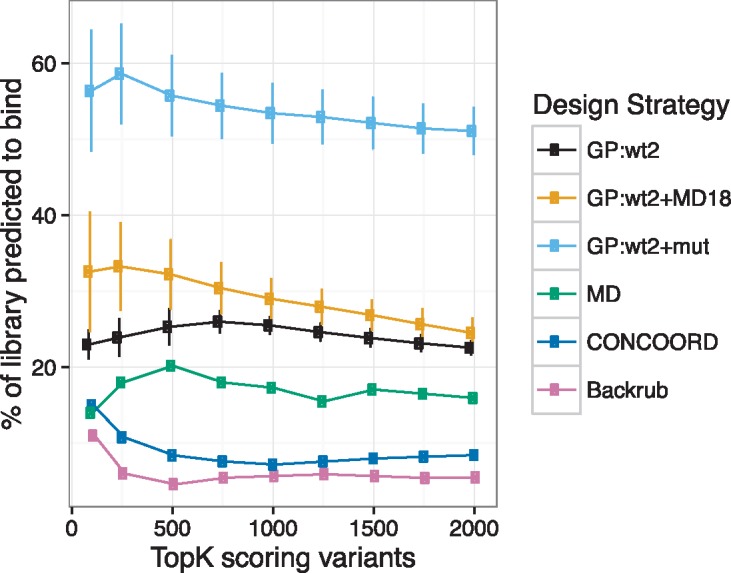
Comparison of protein design strategies. The y-axis reports the number of tight binding variants determined by a random forest regression model. The top scoring ubiquitin variants from each design strategy are selected at thresholds ranging from 100 to 2000. GP:wt2, GP:wt2+MD18, and GP:wt2+mut respectively correspond to the FlexiBaL-GP method being initialized with two wild type structures (3I3T and 2Y5B), two wild type structures and 18 molecular dynamic models, and two wild type structures and a tight binding ubiquitin variant for USP21 (3MTN). MD, CONCOORD, and Backrub designate current ensemble and flexible backbone protein design methods that use single protein structures for the design process.

For emerging approaches that link design and screening, accuracy of “the best” designs is less important (also difficult to achieve) than enrichment of tight binders in a library [20], we hence evaluated the design approaches using this metric. The FlexiBaL-GP approach was found to consistently identify a higher proportion of predicted tight binding ubiquitin variants for USP21 than the ensemble and flexible backbone design methods, as evaluated by our random forest model (Fig 3). Here, predicted tight binding ubiquitin variants are determined with the described random forest regression model. Using just two wild type X-ray crystal structures (3I3T and 2Y5B) as input for FlexiBaL-GP achieved a mean of 451 predicted tight binding ubiquitin variants towards USP21 (23% of the designs), whereas the molecular dynamics ensemble protein design approach identified 319 predicted tight binding ubiquitin variants (16% of the designs, p-value = 0.003069 by a one sample t-test). Augmenting the two wild type X-ray crystal input structures with 18 molecular dynamic models resulted in a mean of 490 tight binding ubiquitin variants (25% of the designs). Current molecular dynamic, CONCOORD, or Backrub design strategies respectively identified 319, 168, and 110 predicted tight binding variants (16.0%, 8.4%, and 5.5% of the designs). Thus, if only wild type information is available, using the FlexiBaL-GP approach with crystal structures augmented with molecular dynamic models would result in 1.5, 2.9, and 4.5 times more tight binding variants when respectively compared to using single template design approaches that incorporate molecular dynamics, CONCOORD, and Backrub strategies (p-value = 0.01386, 0.00142, and 0.00074 by a one sample t-test). Similarly, supplementing the wild type X-ray crystal structures with that of a crystal structure of a tight binding ubiquitin variant for USP21 (3MTN) resulted in a mean of 1022 (51% of the designs) predicted tight binding variants, which is again significantly more than that was discovered by the molecular dynamic ensemble design strategy (p-value = 0.0003684 by a one sample t-test).

**Fig 4:**
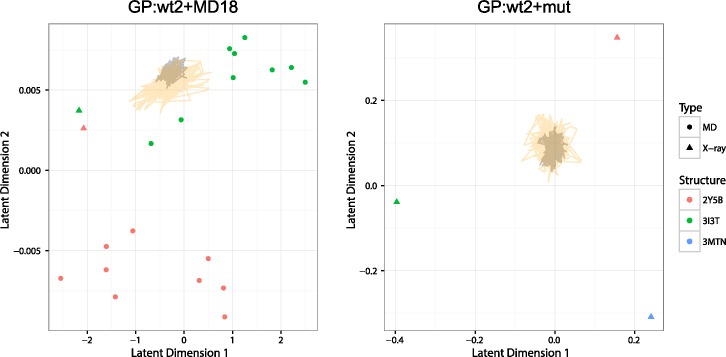
Latent space FlexiBaL-GP trajectory analysis. Representative MCMC trajectories respectively operating at temperatures T = 0.10 (black lines) and T = 0.30 (orange lines) are plotted for two different inputs for the FlexiBaL-GP strategy. Input structures are shown in red, green, and blue, respectively representing structures derived from the 2Y5B, 3I3T, and 3MTN X-ray crystal structures.

**Fig 5:**
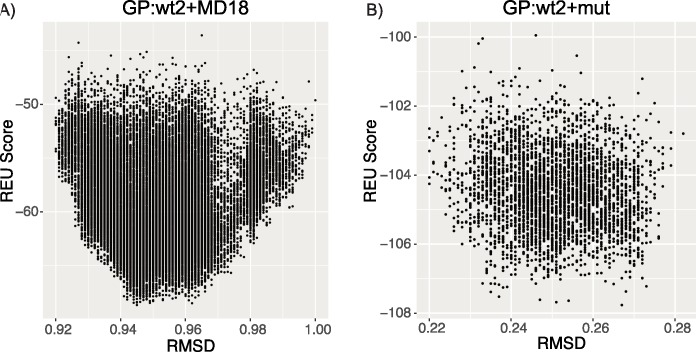
RMSD vs Rosetta energy unit (REU) score for the 10 lowest temperature trajectories of a single FlexiBaL-GP execution for the GP:wt2+MD18 and GP:wt2+mut scenarios.

**Fig 6:**
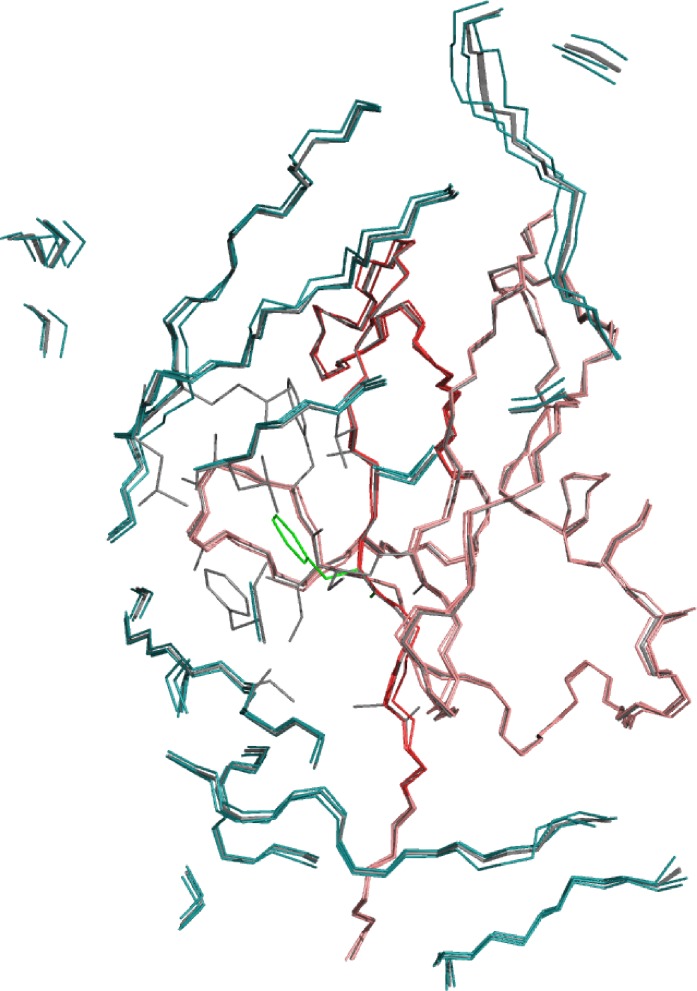
Superimposition of 3 randomly selected FlexiBaL-GP models (gray) from the GP:wt2+mut scenario and the 3 partial X-ray crystal structures used as input. The ubiquitin and USP21 X-ray crystal component is respectively shown in pink and cyan, with the design ubiquitin region colored red. Position 68F from one FlexiBaL-GP model is shown in green.

To ensure that information from multiple protein backbones was utilized during the FlexiBaL-GP protein design process, MCMC trajectories associated with the lowest temperature were inspected. Designing the ubiquitin interface for USP21 binding using two wild type structures augmented with molecular dynamic models as input (GP:wt2+MD18) resulted in the designed ubiquitin variants having protein backbones similar to molecular dynamic models derived from the 3I3T structure (Fig 4). No single input structure was found to determine protein backbones associated with putative tight binding ubiquitin variants. Similarly, augmenting the wild type structures with the 3MTN structure (GP:wt2+mut), a tight binding ubiquitin variant, didn’t result in variants whose backbones were only similar to the 3MTN structure. Rather, information from all three input structures was used to engineer ubiquitin variants for tight binding towards USP21 (Fig 4). These results underline the strength of FlexiBaL-GP to synthesize information from multiple structures. Using the 3I3T partial complex as a reference, low energy FlexiBaL-GP models were found to occupy a specific region in backbone conformational space (Fig 5), corroborating our previous observations. Considering that the RMSD between the 3I3T and 2Y5B X-ray partial structures is 0.449 Å and the RMSD between the 3I3T (wild-type) and 3MTN (mutant) partial structures is 0.421 Å, models associated with the GP:wt2+mut scenario are similar to the input structures (Fig 5). However, since the wild-type and mutant structures are highly similar to each other, the discovery of putative tight binding variants is not reliant on large structural changes but rather on the ability to propose accurate protein backbones that are amendable to putative tight binding variants. Visual inspection of 3 randomly select GP:wt2+mut models superimposed on the 3 input X-ray structures illustrate that the models are highly concordant with the input structures in segments of low variance, whereas in segments of higher variance, the models occupy a different backbone configuration (Fig 6).

**Fig 7:**
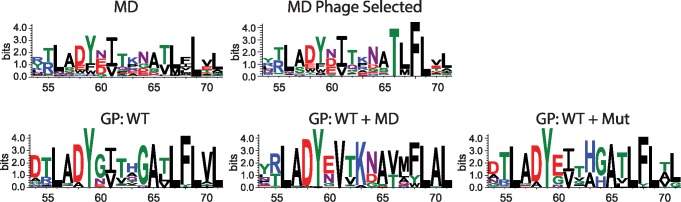
Ubiquitin variant sequence logos. Designed ubiquitin variants using a molecular dynamic (MD) ensemble and validated for USP21 binding by phage display (MD Phage Selected). Variants designed by the FlexiBaL-GP strategy using different input structures: GP:wt2, GP:wt2+MD18, and GP:wt2+mut.

In order to further understand the performance enhancement of using multiple input structures, we compared sequence logos of the designed variants to those of the previously experimentally validated variants [20]. Comparing the best 2000 scoring variants, the FlexiBaL-GP derived variants were found to better recapitulate the tyrosine and phenylalanine at respectively positions 66 and 68 compared to variants from the molecular dynamics protein ensemble design approach (Fig 7). Importantly, the FlexiBaL-GP variants retain the 66T and 68F mutations such that they resemble variants that were experimentally validated by phage display, where 68F enables hydrophobic interactions with USP21 (Fig 6). Additionally, unlike the molecular dynamic ensemble design method, the FlexiBaL-GP approach also identified a tyrosine mutation at position 68 as being important for ubiquitin binding towards USP21, which was previously found in 2 out of 26 variants that were experimentally identified by phage display using a naïve library [32]. Since the employed random forest model used to predict tight USP21 binding was not trained on any variants with the tyrosine mutation, the number of predicted tight binding variants for the FlexiBaL-GP method is likely a lower bound. Furthermore, the amino acid variance across the designed positions for the FlexiBaL-GP variants is less than that observed from the molecular dynamics protein ensemble designed variants, indicating that the FlexiBaL-GP method samples protein variants that are more similar to each other, which are likely close to the optimal sequence conditioned on the scoring function (Fig 7).

**Fig 8:**
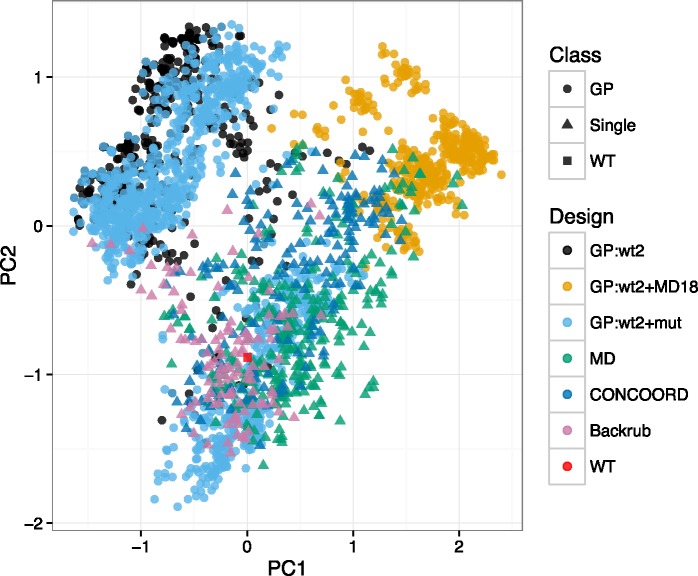
Principal component analysis of designed ubiquitin variants predicted to tightly bind USP21.

Principal component analysis was subsequently employed to explore the sequence variability observed within the FlexiBaL-GP derived variants. We find that variants derived from inputs composed of only X-ray crystal structures (Fig 8, GP:wt2 and GP:wt2+mut) were similar to each other, despite the inclusion of the protein complex of a known ubiquitin variant bound to USP21. Interestingly, inclusion of the 3MTN structure resulted in the appearance of a cluster of protein variants similar to the wild type sequence. Additionally, using multiple protein structures to guide the design process, as is the case for the FlexiBaL-GP method, recovered variants that were distinctly different from variants derived from single protein structure templates. In particular, using a mixture of X-ray structures and molecular dynamic models (GP:wt2+MD18) resulted in the identification of a unique set of variants that differed from other FlexiBaL-GP and single template variants. Furthermore, the designed variants from single template approaches such as ensemble and flexible backbone methods were more similar to the wild type sequence than variants from the FlexiBaL-GP approach (Fig 8).

## Discussion

We have described a new flexible backbone protein design strategy, FlexiBaL-GP design, which leverages information from multiple structures. By learning protein movements directly from multiple protein structures, the FlexiBaL-GP strategy can incorporate large multi-atom movements to explore alternative protein backbones specific to the protein system of interest to identify multiple energetically favourable protein variants. This differs from existing flexible backbone protein design methods that use local rigid perturbations to maintain rigid bond lengths or bond angles to restrict a protein’s degrees of freedom while searching for alternative protein backbone. Flexible backbone methods that use local movements rely on the accuracy of a scoring function to assess the quality of protein backbone structures. On the other hand, structures from FlexiBaL-GP backbone movements are preconditioned to be similar to experimentally determined input protein backbones, minimizing the reliance on a scoring function for the creation of high quality protein backbones. While many protein design methods focus on identifying the optimal sequence, the FlexiBaL-GP method samples protein variants from a target distribution (energy landscape) defined by the scoring function. In this regard it is more akin to the K* dead-end elimination [37], genetic algorithm [21,38], and Backrub methods used for flexible backbone design [23]. The parallel tempering search strategy of the FlexiBaL-GP approach enables it to jointly sample alternative protein backbones and side-chains to identify protein variants that may reside in multiple local minima. The smooth representation of the protein backbone in a lower dimensional space enables the FlexiBaL-GP method to interpolate between known protein structures. It also enables the FlexiBaL-GP strategy to avoid the energy minimization step that current design strategies employ to prepare the input structure for design. While this minimization procedure is used to ensure that the structure is equilibrated with respect to a scoring function, the minimization is performed using the wild-type sequence, which may restrict subsequent designs to be similar to the wild type sequence.

Learning the global backbone movements of a protein system requires multiple protein structures to be provided as input to the FlexiBaL-GP method. Since just a matrix of atomic coordinates are required, X-ray crystal structures, nuclear magnetic resonance (NMR) models, or Cryo–electron microscopy (Cryo-EM) structures can be supplied as input, in addition to computational models. Information from these multiple structures enables the FlexiBaL-GP method to identify 50% more tight binding ubiquitin variants compared to using a molecular dynamics ensemble design strategy, when only wild type structural information is available (Fig 3). If just three X-ray crystal structures are used as input, where one of the structures is a known tight binding variant, 3.2 times the number of tight binding variants can be identified (51% of the designs) compared to using a molecular dynamics ensemble design strategy (16% of the designs). While the large increase in the number of discovered tight binding variants may be attributed to the 3MTN protein backbone predisposing designs for ubiquitin variants that tightly bind USP21, the FlexiBaL-GP method was found to use information from all three X-ray crystal structures (Fig 4). This suggests that the increased performance arises from the addition of general structural constraints being placed on the space of discoverable protein backbones during the design process. Thus unlike existing methods, the FlexiBaL-GP approach can be used to iteratively improve upon existing design solutions by alternating between computational design and incorporating additional solved protein structures.

Here, we have described the application of the GP-LVM dimensionality reduction method for protein design within our FlexiBaL-GP protein design method. The GP-LVM model can also be used for the analysis of molecular dynamic trajectories or to infer possible structural configurations along a reaction pathway. For the purposes of protein design, the GP-LVM enables the recovery of more tight binding variants than existing protein design methods that primarily use information from a single structural template to infer new protein backbones. Thus as more structures are deposited into the PDB, our FlexiBaL-GP flexible backbone strategy will only continue to improve, as the space of valid protein backbones is refined with additional structural data. With the introduction of high throughput screening strategies of computational protein designs for binding [20], our flexible backbone design strategy will enable better utility of the screening library for the identification of variants possessing the function of interest.

## Methods

### Ensemble and flexible backbone protein design

Protein design of the ubiquitin interface for USP21 was performed using the Rosetta software suite version 3.5 (build 2013, week 42). Input for the ensemble and flexible backbone protein design strategies originated from Human ubiquitin—USP21 complexes (PDB id 3I3T, 2Y5B, 3MTN) retrieved from the PDB. Before design, each chain was minimized with the Rosetta relax program with the parameters “-relax:constrain_relax_to_start_coords -relax:coord_constrain_sidechains -relax:ramp_constraints false -ex1 -ex2 -use_input_sc -flip_HNQ -no_optH false -dun10 true -score:weights talaris2013”.

Performing ensemble and flexible backbone design with the Rosetta design software for the ubiquitin–USP21 system has been previously described [20]. Briefly for ensemble design, ensembles were generated from the CONCOORD distance constraints method [36] and 100ns molecular dynamics simulations using the GROMACS package [34,35] with the AMBER forcefield [39]. In order to perform the molecular dynamic simulations, missing loops were complete by the FALC-Loop method [40]. The Rosetta multi-cooling simulated annealing approach was used for protein design with the parameters “-score:weights talaris2013 -no_his_his_pairE -extrachi_cutoff 0 -multi_cool_annealer 10”. Flexible backbone design as implemented by the Rosetta Backrub application was executed with the flags “-ex1 -ex2 -dun10 true -score:weights talaris2013 -extrachi_cutoff 0 -no_his_his_pairE -backrub:ntrials 10000”.

### FlexiBaL-GP protein design

Protein backbone heavy atoms were first extracted and aligned using the Kabsch algorithm [41] as implemented in the R Project bio3d library [42]. For the input scenario utilizing molecular dynamic simulations, 9 representative models were selected from each 3I3T and 2Y5B trajectory. Representative models were selected by average-link clustering of the molecular dynamic models. Models from before the trajectory stabilized were removed and the model closest to a cluster’s average was chosen as a representative. These 18 representative molecular dynamic models were combined with the 2 wild type structures to give a total of 20 structures. In all other cases, atomic coordinates directly from the experimentally determined structure are used. The GPy framework [30] is subsequently used to learn the GPLVM parameters arising from using the composite RBF + Bias kernel. For all scenarios in this study, the FlexiBaL-GP design process was executed for 2 million iterations, with models being saved to disk every 200 iterations. The first 20 000 and 600 000 iterations are considered burn-in for the FlexiBaL-GP trajectories respectively for the ubiquitin ensemble and ubiquitin–USP21 design scenarios. The size of the latent space step is determined by dividing the distance between the maximum and minimum values of a latent dimension by 1000. At each iteration, a side chain is chosen 95% of the time, whereas a protein backbone movement is chosen 5% of the time. In particular, the Rosetta SidechainMover is used for the side-chain selection process. 24 MCMC trajectories were used, which were spaced over a temperature ladder defined by a geometric series with a multiplying ratio of 1.1 between the temperatures 0.1 and 0.5 inclusively.
